# Assessing the complexity of interventions within systematic reviews: development, content and use of a new tool (iCAT_SR)

**DOI:** 10.1186/s12874-017-0349-x

**Published:** 2017-04-26

**Authors:** Simon Lewin, Maggie Hendry, Jackie Chandler, Andrew D. Oxman, Susan Michie, Sasha Shepperd, Barnaby C. Reeves, Peter Tugwell, Karin Hannes, Eva A. Rehfuess, Vivien Welch, Joanne E. Mckenzie, Belinda Burford, Jennifer Petkovic, Laurie M. Anderson, Janet Harris, Jane Noyes

**Affiliations:** 1 Norwegian Institute of Public Health, Oslo, Norway; 2 Health Systems Research Unit, South African Medical Research Council, Cape Town, South Africa; 3 North Wales Centre for Primary Care Research, Bangor University, Bangor, UK; 4 Cochrane, Cochrane Central Executive, London, UK; 5 Centre for Behaviour Change, University College London, London, UK; 6 Nuffield Department of Population Health, University of Oxford, Oxford, UK; 7 Clinical Trials and Evaluation Unit, School of Clinical Sciences, University of Bristol, Bristol, UK; 8 Institute of Population Health, University of Ottawa, Ottawa, Canada; 9 Social Research Methodology Group, Centre for Sociological Research, Faculty of Social Sciences, KU Leuven, Leuven, Belgium; 10 Institute for Medical Informatics, Biometry and Epidemiology, University of Munich, Munich, Germany; 11 Bruyère Research Institute, Bruyère Continuing Care, Ottawa, Ontario Canada; 12 School of Epidemiology, Public Health and Preventive Medicine, University of Ottawa, Ottawa, Canada; 13 Ottawa Hospital Research Institute, Ottawa, Ontario Canada; 14 School of Public Health and Preventive Medicine, The Alfred Centre, Monash University, Melbourne, Victoria Australia; 15 Cochrane Public Health Group and Jack Brockhoff Child Health and Wellbeing Program, Melbourne School of Population and Global Health, University of Melbourne, Melbourne, Victoria Australia; 16 Campbell and Cochrane Equity Methods Group, Centre for Global Health, Institute of Population Health, University of Ottawa, Ottawa, Canada; 17 Department of Epidemiology, School of Public Health, University of Washington, Seattle, USA; 18 School of Health and Related Research (ScHARR), University of Sheffield, Sheffield, UK; 19 School of Social Sciences, Bangor University, Bangor, UK

**Keywords:** Complex interventions, Systematic review, Tool, Intervention development, Evidence synthesis, Complex, Intervention, Complexity

## Abstract

**Background:**

Health interventions fall along a spectrum from simple to more complex. There is wide interest in methods for reviewing ‘complex interventions’, but few transparent approaches for assessing intervention complexity in systematic reviews. Such assessments may assist review authors in, for example, systematically describing interventions and developing logic models. This paper describes the development and application of the intervention Complexity Assessment Tool for Systematic Reviews (iCAT_SR), a new tool to assess and categorise levels of intervention complexity in systematic reviews.

**Methods:**

We developed the iCAT_SR by adapting and extending an existing complexity assessment tool for randomized trials. We undertook this adaptation using a consensus approach in which possible complexity dimensions were circulated for feedback to a panel of methodologists with expertise in complex interventions and systematic reviews. Based on these inputs, we developed a draft version of the tool. We then invited a second round of feedback from the panel and a wider group of systematic reviewers. This informed further refinement of the tool.

**Results:**

The tool comprises ten dimensions: (1) the number of active components in the intervention; (2) the number of behaviours of recipients to which the intervention is directed; (3) the range and number of organizational levels targeted by the intervention; (4) the degree of tailoring intended or flexibility permitted across sites or individuals in applying or implementing the intervention; (5) the level of skill required by those delivering the intervention; (6) the level of skill required by those receiving the intervention; (7) the degree of interaction between intervention components; (8) the degree to which the effects of the intervention are context dependent; (9) the degree to which the effects of the interventions are changed by recipient or provider factors; (10) and the nature of the causal pathway between intervention and outcome. Dimensions 1–6 are considered ‘core’ dimensions. Dimensions 7–10 are optional and may not be useful for all interventions.

**Conclusions:**

The iCAT_SR tool facilitates more in-depth, systematic assessment of the complexity of interventions in systematic reviews and can assist in undertaking reviews and interpreting review findings. Further testing of the tool is now needed.

**Electronic supplementary material:**

The online version of this article (doi:10.1186/s12874-017-0349-x) contains supplementary material, which is available to authorized users.

## Background

‘Complex interventions’ in health have been debated widely for several years. Key points of discussion include how to define such interventions, how to evaluate their effects and how to synthesise these effects within systematic reviews [1–5]. Some common features of complex interventions in health identified some years ago by the UK Medical Research Council (MRC) and widely quoted [6] include: having a number of components that may act both dependently and independently; having “active ingredients” that are not easy to define; being interventions that may be delivered at the individual, organisational or population level; and being targeted towards patients directly or indirectly through health professionals or health systems.

There are many other definitions of complex interventions, often highlighting multiple, interacting components and non-linear causal pathways, and emphasising variability in content, context and mode of delivery, as well as the unpredictability of their effects [2, 7, 8]. Other approaches have sought to differentiate complex, complicated and simple interventions [9, 10]. For example, it has been suggested that interventions with many component parts that may require a high level of skill in delivery but have little variation in application or outcome are ‘complicated’ rather than ‘complex’, and that interventions with few components that can be delivered in a formulaic manner with predictable outcomes are ‘simple’. Complex interventions or systems, in contrast, have features of non-linearity, context dependency, adaptability and interdependence of intervention elements [11]. Further work has focused on ways to graphically depict complex interventions, including how they are delivered [12, 13]. While useful, these approaches are not focused on understanding intervention complexity in the context of systematic reviews. Furthermore, intervention complexity has many dimensions, and interventions may show more complexity on some of these dimensions than on others. It is therefore difficult and probably not useful to create a simple definition of what we might view as a complex intervention. Instead, the tool described in this paper outlines the key dimensions of intervention complexity and describes how these might be assessed. Using the tool, interventions can be described as having degrees of complexity in relation to these dimensions.

In other strands of methodological work, we have proposed a new conceptual approach to categorising and describing intervention complexity [14]. This approach distinguishes between the description and analysis of: a) intervention complexity (i.e. situations in which we expect the effects of an intervention to be modified by variant properties or characteristics of the intervention itself); b) complexity in implementation (i.e. situations in which we expect the effects of an intervention to be modified by variant characteristics of implementation processes); c) complexity in context (i.e. situations in which we expect the effects of an intervention to be modified by variant properties or characteristics of the settings or contexts in which an intervention is implemented); and d) complexity in relation to participants’ characteristics (i.e. situations in which we expect the effects of an intervention to be modified by variant characteristics of participants receiving an intervention). This approach has informed the development of the tool described in this paper.

Being able to describe and replicate interventions that are seen as lying towards the more complex end of the simple-complex continuum is important because of the wide use of such interventions in health and social care and because of the growing interest in understanding both their effectiveness and how these effects come about [15]. Systematic review methods are frequently used to quantify these effects. However, to be useful to those deciding whether and how to implement interventions, such reviews also require precise descriptions of the interventions [16] and an understanding of why an intervention should be considered complex. Describing and assessing these interventions is challenging though because they may have many interacting components and because they need to be described and assessed in relation to how they are implemented, the context in which they are implemented and those delivering and receiving the intervention [15]. Clark [9] argues that research into complex health interventions still focuses on easily described components of interventions and risks overlooking the key issue of which components are most strongly associated with the effects of the intervention. In other words, ways of describing complex interventions need to help us ultimately in understanding their mechanisms of action and effects – how and why they work in a given context.

The MRC guidance for developing and evaluating complex interventions, updated in 2008 [6, 17], has been influential in taking forward this field but conducting systematic reviews that fully address the complexity of interventions remains challenging [3, 14, 18]. In particular, systematic reviews need to be able to describe complexity accurately and consistently across included primary studies in order to facilitate the grouping of studies for analysis, investigate causes of heterogeneity, and explore the relationship between intervention complexity and fidelity of implementation [19, 20]. The new approach to categorising intervention complexity described above [14] may help authors understand the contextual considerations contributing to intervention mechanisms and effects [3, 21]. In addition, an approach has been developed for identifying key intervention content and implementation processes [22]. However, currently there is no widely accepted and used tool or typology for assessing and categorizing levels of intervention complexity.

This paper describes the development of the intervention Complexity Assessment Tool for Systematic Reviews (iCAT_SR). The tool aims to provide an overall picture of the complexity of an intervention that can be depicted visually, for example in a chart, or contribute to developing or refining a logic model. In addition, the tool may contribute to understanding intervention heterogeneity and informing decisions about the implementation of effective interventions.

### Aim

To describe the development and application of the iCAT_SR tool to assess and categorise levels of intervention complexity in systematic reviews.

## Methods

We developed the iCAT in two stages: in the first stage, we developed an initial version of the tool to assess intervention complexity in randomised trials [7]. The steps we undertook in developing this tool are detailed in Additional file 1. The output of this stage comprised a guidance document describing six dimensions of complexity (Additional file 2) and a template for rating each dimension using a system with three levels. We considered and excluded a further ten dimensions, mainly because they were already covered by an included dimension, were thought too difficult to assess objectively or pre-trial, or did not to relate directly to the complexity of an intervention (Additional file 3).

In the second stage, we adapted and further developed the existing iCAT tool so that it could be used to describe and assess intervention complexity in systematic reviews. We undertook the following steps:

- *Consensus approach:* We circulated the iCAT dimensions identified as being relevant to describing the complexity of interventions in single, primary evaluation studies [7] (Additional file 2) for feedback and comment to an expert panel of methodologists working in the areas of complex interventions and systematic reviews. Through a series of group and individual telephone calls, we obtained feedback on whether the iCAT tool could be adapted for application in systematic reviews of complex interventions; and how best to integrate the tool into the review process. A total of 18 people participated in the discussions and/or provided feedback on the tool. We then collated and circulated all comments for confirmation of feedback and also invited additional comments.
- *Drafting of first version of the iCAT_SR:* Based on the feedback received, we revised the iCAT for single studies to include four additional dimensions seen to be useful in the context of systematic reviews. These were: (1) the degree of interaction between intervention components; (2) the degree to which the effects of the intervention are dependent on the context or setting in which it is implemented; (3) the degree to which the effects of the intervention are changed by recipient or provider factors; and (4) the nature of the causal pathway between the intervention and the outcome it is intended to effect.
- *Second round of feedback on the draft iCAT_SR:* We circulated a draft version of the tool, incorporating the agreed amendments, among the expert panel as well as to a wider group of systematic reviewers with an expressed interest in complex interventions. We approached the latter through a ‘complex intervention’ e-mail list. We then invited all those who had contributed to join a meeting at the 2012 Cochrane Colloquium (which around 50 people attended), where the development work was presented and the tool demonstrated through application to two empirical examples. Group discussion following the presentation resulted in additional feedback which informed further refinement of the tool.

## Results

### Dimensions of the iCAT_SR version 1

The revised tool comprises six ‘core’ and four ‘optional’ dimensions for assessing intervention complexity. The core dimensions, which include those in the original iCAT tool, and the optional dimensions, seen to be useful in the context of systematic reviews specifically, are described briefly below and in more detail in Tables 1 and 2. A detailed description of the tool and guidance on how to apply it can be found in Additional file 4.

**Table 1:** Summary of core dimensions and assessment criteria for the iCAT_SR

Core dimension	Assessment levels and criteria for each dimension	
1. Active components included in the intervention, in relation to the comparison	*More than one component and delivered as a bundle*	The intervention includes more than one component and some or all of these components need to be delivered as a bundle.
	*More than one component*	The intervention includes more than one component. These components may be integrated into a package.
	*One component*	The intervention includes one component only.
	*Varies* ^a^	Varies across interventions to be considered for/included in the review.
2. Behaviour or actions of intervention recipients or participants to which the intervention is directed	*Multi-target*	Intervention directed at three or more behaviours or actions.
	*Dual target*	Intervention directed at two behaviours or actions.
	*Single target*	Intervention directed at one behaviour or action only.
	*Varies* ^a^	Varies across interventions to be considered for/included in the review.
3. Organisational levels and categories targeted by the intervention	*Multi-level*	Intervention directed at two or more levels.
	*Multi-category*	Intervention directed at two or more categories of individuals within the individual level (e.g. primary care professionals and primary care patients).
	*Single category*	Intervention directed only at single category of individuals within the individual level (e.g. professionals or patients or policy makers).
4. The degree of tailoring intended or flexibility permitted across sites or individuals in applying or implementing the intervention	*Highly tailored/flexible*	High degree of variation in implementation from site to site permitted and/or intervention designed to tailor to individuals or specific implementation settings.
	*Moderately tailored/flexible*	Some variation in implementation from site to site permitted (i.e. some components of the intervention are tailored/flexible while others are not).
	*Inflexible*	Intervention implementation highly standardised with minimal variation from site to site.
	*Varies* ^a^	Varies across interventions to be considered for/included in the review
5. The level of skill required by those delivering the intervention in order to meet the intervention objectives	*High level skills*	Extensive specialised skills required, i.e. new skills in addition to expected existing skills AND/OR the extension of existing skills to a highly specialised area AND/OR skills requiring extensive additional training.
	*Intermediate level skills*	Some specialised skills required, i.e. a small extension to the expected existing skills of professionals, decision makers or consumers.
	*Basic skills*	No specialised skills required.
	*Varies* ^a^	Varies across interventions to be considered for/included in the review.
6. The level of skill required for the targeted behaviour when entering the included studies by those receiving the intervention, in order to meet the intervention objectives	*High level skills*	Extensive specialised skills required.
	*Intermediate level skills*	Some specialised skills required.
	*Basic skills*	No specialised skills required.
	*Varies* ^a^	Varies across interventions to be considered for/included in the review.

^a^If this category is selected, review authors should consider whether the interventions included in the review are as similar as originally thought and whether this has implications for the review’s inclusion criteria

**Table 2:** Summary of optional dimensions and assessment criteria for the iCAT_SR

Optional dimension	Assessment levels and criteria for each dimension	
7. The degree of interaction between intervention components, including the independence/interdependence of intervention components	*High level interaction*	There is substantial interaction or inter-dependency between intervention components or actions i.e. the delivery of one intervention component impacts on the delivery of another, resulting in a synergistic effect.
	*Moderate interaction*	There is some degree of interaction but no evidence of synergistic effects or dysynergistic effects.
	*Independent*	The intervention has only one component or action, or the components act independently.
	*Varies* ^a^	Varies across interventions to be considered for/included in the review.
	*Unclear or unable to assess*	
8. The degree to which the effects of the intervention are dependent on the context or setting in which it is implemented	*Highly context dependent*	The effects of the intervention are likely to be strongly dependent on the implementation setting.
	*Moderately context dependent*	The effects of the intervention are likely to be transferrable across a limited range of settings only (e.g. only within a specific country or health system).
	*Independent of context*	The effects of the intervention do not appear to be strongly dependent on the implementation setting, i.e. it is anticipated that the effects of the intervention will be similar across a wide range of contexts or settings.
	*Varies* ^a^	Varies across interventions to be considered for/included in the review.
	*Unclear or unable to assess*	
9. The degree to which the effects of the intervention are changed by recipient or provider factors	*Highly dependent on individual-level factors*	The effects of the intervention are modified by both recipient and provider factors.
	*Moderately dependent on individual-level factors*	The effects of the intervention are modified by one of recipient or provider factors.
	*Largely independent of individual-level factors*	The effects of the intervention are not modified substantially by recipient or provider factors.
	*Varies* ^a^	Varies across interventions to be considered for/included in the review.
	*Unclear or unable to assess*	
10. The nature of the causal pathway between the intervention and the outcome it is intended to effect	*Pathway variable, long*	The causal pathway includes three or more steps between intervention and outcome or occurs over a long time period; is not linear, or is variable; and/or more than one causal pathway has been proposed.
	*Pathway linear, long*	The causal pathway is linear but there are three or more steps between intervention and outcome.
	*Pathway linear, short*	The causal pathway is clear, short (only one or two steps), direct, linear.
	*Varies* ^a^	Varies across interventions to be considered for/included in the review.
	*Unclear or unable to assess*	

^a^If this category is selected, review authors should consider whether the interventions included in the review are as similar as originally thought and whether this has implications for the review’s inclusion criteria

Core dimensions:

1. ***Active components*** *included in the intervention, in relation to the comparison:* An intervention component is defined as a discrete, active element of the intervention that could be implemented independently of other elements. Components vary in number and could be delivered independently of each other or be grouped together in organised bundles or looser packages of care.
2. ***Behaviour*** *or actions* ***of*** *intervention* ***recipients*** *or participants to which the intervention is directed:* Behaviours or actions include taking a medication, changing a particular practice, improving knowledge or undergoing a surgical procedure; they may also include *not* undertaking a behaviour, such as not smoking. Behaviours or actions are targeted by the active components of the intervention.
3. ***Organisational levels*** *and categories targeted by the intervention:* Level refers to whether the intervention was directed at individuals (consumers, professionals, policy makers); groups or teams of individuals (staff of clinics, patient support groups, surgical teams etc.); or systems (communities, health systems, organisations (such as hospitals), policy networks). Categories are groups, such as nurses or patients, within those levels.
4. *The* ***degree of tailoring intended*** *or flexibility permitted across sites or individuals in applying or implementing the intervention*: Tailoring implies that the intervention is intended to be modified for specific individuals, settings or circumstances, whereas flexibility implies leeway for modification if desired. Interventions may be modifiable in both content (e.g. variation in the components received by sites or individuals) and form (variation in the ways in which the components are delivered across sites or individuals).
5. *The level of* ***skill required by those delivering*** *the intervention in order to meet the intervention objectives:* Skill is defined as the ability to do something, such as deliver a health promotion message appropriately or provide supportive supervision to health workers, arising from training, practice or experience. Different levels of skills may be required to deliver different interventions.
6. *The level of* ***skill required*** *for the targeted behaviour when entering the included studies* ***by those receiving*** *the intervention (consumers, professionals, planners) in order to meet the intervention objectives:* Those receiving an intervention, such as an educational programme, may need skills, based on training, experience or practice, to interpret the information provided and then to apply it in their setting. For example, consumers may need a certain level of internet and heath literacy to access and use health information. Again, different levels of skills may be required for different interventions.

Optional dimensions:

7. *The degree of* ***interaction between intervention components*** *, including the independence/interdependence of intervention components:* The effectiveness of an intervention may depend on the combination of components delivered and/or the sequence of delivery. There may be synergistic (“added value”) or dysynergistic effects from delivering intervention components in a particular combination, and one component delivered alone could be effective, ineffective or even harmful (Table 3).

**Table 3:** Interactions and interdependencies within complex interventions (adapted from [40, 41])

Complex interventions can include components that interact synergistically or dysynergistically, as follows: • Synergistic: Intervention components interact in ways that the total effect is greater than the sum of the individual effects of the components. • Dysynergistic: Intervention components act in ways that the total effect is less than the sum of the individual effects of the components. Where intervention components do not interact in these ways, one would expect the effect of the intervention to be the sum of the individual effects of all of the components. Complex interventions can also include components that are interdependent. Where such interdependencies exist, they can be described as: • Contemporaneous: The effect of one intervention component depends on another intervention component being present at the same time. On their own, each component may be less effective, ineffective, or harmful. • Temporal: The effect of one intervention component depends on another component being present beforehand. On their own, each component may be less effective, ineffective, or harmful. Where intervention components do not show interdependency, one would expect these components to be effective regardless of the presence or absence of other components.
8. *The degree to which the effects of the intervention are* ***dependent on the context or setting*** *in which it is implemented:* The effects of an intervention may be dependent on the societal, political, economic, health system or environmental context in which the intervention is delivered. For example, an intervention may not have the same effects in primary care clinics and tertiary level hospitals, or in a health system in which care is free at the point of contact compared to one in which that is not the case.
9. *The degree to which the effects of the* ***intervention*** *are* ***changed by recipient or provider factors*** *:* The effects of an intervention may be dependent on the recipient’s readiness for behaviour change or the proficiency of the person delivering the intervention.
10. *The* ***nature of the causal pathway*** *between the intervention and the outcome it is intended to effect:* This refers to pathways that involve human actions (such as behaviours) or actions within organisations or systems rather than biological pathways. The causal pathway for an intervention may be clear, direct, short and linear or it may be longer, more variable, or there may be more than one causal pathway [23–25].

### Grading the complexity of interventions using iCAT_SR

For each iCAT_SR dimension an intervention can be graded on one of three levels, ranging from more simple to more complex (Tables 1 and 2). For some components, it is also possible to select ‘varies’ where that particular component varies across interventions to be considered for the review or ‘unclear or unable to assess’ when the information needed to make an assessment is not available. Review authors should provide support for their judgements regarding these assessments so as to improve transparency and help readers understand the judgements made (Additional file 4). Information for the support for judgement may be drawn from multiple sources: published study reports; ancillary papers on the studies, including qualitative process evaluations; and information obtained from study authors. Where a review of effectiveness has a linked qualitative evidence synthesis that explores how the intervention works and factors affecting its implementation, this additional information may be very helpful in making assessments for the complexity dimensions. This is shown in the example in Table 4 which draws on data from both a Cochrane review of effectiveness [26] and a Cochrane qualitative evidence synthesis [25].

**Table 4:** Applying the iCAT_SR – example A^a^

Core dimension	Description of the intervention in the review	Judgement	Support for judgement
1. Active components included in the intervention, in relation to the comparison	‘Any intervention delivered by LHWs [lay health workers] and intended to improve maternal or child health (MCH) or the management of infectious diseases.’ ([26] p7)	*One component*	The active component is the delivery by a LHW of a health intervention. Although the nature of the intervention delivered and the extent to which LHWs worked with other providers varied considerably across trials included in the review, all interventions were delivered by LHWs.
2. Behaviour or actions of intervention recipients or participants to which the intervention is directed	‘Any intervention delivered by LHWs and intended to improve maternal or child health (MCH) or the management of infectious diseases…[]…a MCH or infectious diseases intervention was defined as follows. • Child health: any interventions aimed at improving the health of children aged less than five years. • Maternal health: any interventions aimed at improving reproductive health, ensuring safe motherhood, or directed at women in their role as carers for children aged less than five years. • Infectious diseases: any interventions aimed at preventing, diagnosing, or treating communicable diseases…’ ([26] p7-8)	*Varied*	Included interventions varied from having a single target (e.g., initiation of breastfeeding) to having multiple targets (e.g., community-based interventions directed at hygiene practices, nutrition practices and child caring behaviours among recipients, and intended to reduce neonatal mortality).
3. Organisational levels and categories targeted by the intervention	‘There were no restrictions on the types of patients or recipients for whom data were extracted.’ ([26] p7)	*Single category*	The interventions delivered by LHWs were directed at individual patients or community members, or groups of patients or community members, within communities or primary care.
4. The degree of tailoring intended or flexibility permitted across sites or individuals in applying or implementing the intervention	‘Any intervention delivered by LHWs and intended to improve maternal or child health (MCH) or the management of infectious diseases.’ ([26] p7)	*Varied from inflexible to highly flexible*	Because the review included any intervention delivered by LHWs and intended to improve MCH or the management of infectious diseases, the range of included interventions was very wide. Some interventions were implemented in a highly standardised way (e.g., structured telephone support for pregnant women from high risk groups [42]) while others allowed variation from site to side or individual tailoring (e.g., provision of health and parenting education to inner city mothers [43]).
5. The level of skill required by those delivering the intervention in order to meet the intervention objectives	‘Any lay health worker (paid or voluntary) …[]…For the purposes of this review, we defined the term lay health worker as any health worker who: • performed functions related to healthcare delivery, • was trained in some way in the context of the intervention, but • had received no formal professional or paraprofessional certificate or tertiary education degree.’ ([26] p7)	*Mostly varied from basic to intermediate level skills*	In the studies included in the review, all of the participating LHWs would have received some level of training. In some studies, LHWs received additional training to extend their skills so that they could deliver a specific task or tasks.
6. The level of skill required for the targeted behaviour when entering the included studies by those receiving the intervention, in order to meet the intervention’s objectives	‘There were no restrictions on the types of patients or recipients for whom data were extracted.’ ([26] p7)	*Basic skills*	No specialised skills were required of the patients/consumers participating in the trials.
Optional dimension	Description of the intervention in the review	Judgement	Support for judgement
7. The degree of interaction between intervention components, including the independence/interdependence of intervention components	The degree of interaction between intervention components was not specified in the review inclusion criteria, described explicitly in the data extraction or analysed as part of the review. The intervention was considered to have only one component for the purpose of the review.	*Unclear or unable to assess*	Not described or analysed in the review. Likely to vary across the included studies.
8. The degree to which the effects of the intervention are dependent on the context or setting in which it is implemented	‘A substantial proportion of the included studies…were conducted in LMICs [low and middle income countries] or were directed at low income groups in high income countries. Based on the premise that low income groups across different countries share similar constraints in accessing health care, it may be concluded that these interventions could potentially be extrapolated to other settings, be effective in reaching low income groups, and contribute to reducing health inequalities. However, the degree to which the findings from studies in high income settings can be generalised to low income settings remains unclear and requires further empirical research.’ ([26] p49) ‘While we explored whether there were differences between high, middle and low income countries in the barriers and facilitators we identified, the differences we did find were perhaps surprisingly few…[]… Some differences between settings did emerge, however.’ ([25] p39)	*Moderately to highly context dependent*	The effectiveness review did not address this question but identified it as important to consider in future work. The qualitative evidence synthesis noted that descriptions of study context were limited. The broad categories of high, middle and low income country did not appear to be key in terms of context dependency, but the synthesis identified a wide range of other ways in which the effects of LHW programmes may be dependent on implementation context or setting.
9. The degree to which the effects of the intervention are changed by recipient or provider factors	Not considered in detail in the reviews.	*Moderately to highly dependent on individual-level factors*	Many LHW interventions are intended to change the behaviour or recipients (e.g. to increase breastfeeding or promote adherence to a treatment). We would therefore expect these interventions to be dependent on recipients’ readiness for behaviour change, their self-efficacy and the social support that they receive.
10. The nature of the causal pathway between the intervention and the outcome it is intended to effect	‘…the findings of the qualitative review were organised into chains of events that we proposed could lead to the outcomes measured in the review of effectiveness…’ ([25] p35).	*Pathway variable, long*	More than one causal pathway was identified and each pathway included three or more steps between intervention and outcome.

^a^Drawn from systematic reviews of lay health worker (LHW) interventions in primary and community health care for maternal and child health and the management of infectious diseases [25, 26]

At the development and protocol stages of a review, review authors may not have sufficient information to make definitive judgements regarding a dimension. In these cases, we recommend that the review team make a provisional judgement or develop a hypothesis and then note these hypotheses in their support for judgement. Information emerging at the analysis stage, such as the results of subgroup analyses, may allow judgements to be made with greater confidence. The iCAT_SR assessment can then be amended. This is discussed in more detail below.

### Using the iCAT_SR in systematic reviews of effectiveness

Use of the iCAT_SR in the context of systematic reviews of effectiveness is intended to facilitate a more systematic and thorough understanding of intervention complexity, including in relation to both the content of an intervention and its mode of delivery. Table 5 shows how the tool may be useful at different stages in the process of conducting a systematic review, including in protocol development, analysis and in interpreting and presenting the review findings. For instance, the tool could be used to compare the complexity characteristics of the included studies with those specified using iCAT_SR at the protocol stage. This may help to identify areas in which the intervention description needs to be improved for the next review update. Examples of how iCAT_SR can be applied within systematic reviews are provided in Tables 4 and 5 and Additional file 5. The example in Table 4 focuses on a health systems intervention [25, 26] while that in Additional file 5 focuses on a public health intervention directed to consumers and communities [27, 28].

**Table 5:** Using the iCAT_SR in systematic reviews of the effectiveness of interventions

Stage in the review process^a^	Utility of the iCAT_SR
*Formulating the PICO review question and developing criteria for including studies*	Prompts review authors to identify the key components of the intervention/s and how these interact; the actions to which these components are directed; the organisational levels targeted; the anticipated causal pathway/s or logic model etc. Overall, this may help review authors to conceptualise the intervention and define the scope of the review.
*Searching for studies*	By prompting to review authors to identify the key components of intervention/s and the recipients and organisational levels targeted, the tool may aid in identifying appropriate search terms. This may help in identifying eligible studies where, for example, the interventions of interest are broadly similar in terms of their component parts but have widely varying names in the literature.
*Selecting studies for inclusion*	Makes explicit the key components of the intervention/s and the recipients and organisational levels targeted, and therefore helps to ensure that study inclusion decisions are easier and more consistent across the review author team.
*Extracting data*	Facilitates the organisation and standardisation of data relating to intervention description and intervention complexity. The dimensions of the tool can inform development of the data extraction form for the review.
*Analysing data and undertaking meta-analyses*	Enables classification or grouping of interventions for analysis based on their components and/or participants and levels targeted. The tool may also inform analyses and interpretation by helping to generate *a priori* hypotheses about explanatory factors that could potentially explain differences in results both across studies and across subgroups within studies. These explanatory factors can then be used to explore heterogeneity in subgroup analyses and meta-regressions.
*Presenting results and developing ‘Summary of findings’ tables* ^b^	Enables classification or grouping of interventions based on their components and/or participants and levels targeted, and thus facilitates clear and logical presentation of the review findings. The tool may also identify important research gaps, for example where the causal pathway of an intervention is not clear or where there are important questions regarding interactions between intervention components.
*Interpreting results and drawing conclusions*	Aids refining a logic model or causal pathway for the intervention/s that was developed at the protocol stage.

^a^The stages in this table are based on those for Cochrane reviews, but are also relevant to most other reviews of the effectiveness of interventions

^b^A summary of findings table shows the quality of evidence and magnitude of relative and absolute effects for each outcome in a review assessed as important by stakeholders [44]

At the development and protocol stages of a review, the iCAT_SR can prompt review authors to identify the dimensions of complexity that are associated with a particular intervention. This may improve review authors’ understanding of how to conceptualise and define the intervention they are considering. This, in turn, may improve their description of the intervention in the review protocol (which could follow the format used in Table 4 and Additional file 5) and the identification of eligible studies. As noted above, review authors may select ‘varies’ where a particular iCAT_SR component varies across the group of interventions to be considered for a review. This grading should prompt review authors to consider whether it is appropriate for these interventions to be grouped together in a single review and also, where relevant, whether these interventions would be better considered as separate intervention-comparison combinations within a review. Where a review includes more than one intervention-comparison combination, it is may be appropriate to undertake separate iCAT_SR assessments for each combination.

At the development stage of a review, applying the iCAT_SR may also help identify possible explanatory factors for differences in results across studies and subgroups, and these can then be specified for *a priori* subgroup analyses. For instance, the iCAT_SR example in Table 4 notes, based on findings from a linked qualitative evidence synthesis, that the effects of some lay health worker interventions may be modified by the degree of social support that lay health workers receive. This might therefore be an important explanatory factor to include in subgroup analyses in a systematic review of the effects of lay health worker interventions.

We recognise that limited knowledge of how an intervention works may reduce review authors’ ability to complete the assessment for each complexity dimension at the protocol stage, and that these assessments are a reflexive process involving judgements. We recommend that review authors complete the assessments as far as possible, based on existing knowledge, including from sources such as linked qualitative evidence syntheses, or logical argument and then consider during the analysis stage of the review whether any changes are needed. For example, the findings of a systematic review of intervention effectiveness or of a linked qualitative evidence synthesis may suggest that an intervention-effects causal pathway hypothesized at the protocol stage was incorrect or incomplete. Applying the iCAT_SR might also influence decisions on when to incorporate qualitative evidence to explain gaps in knowledge.

The iCAT_SR is designed to be applied in reviews of a single intervention or of a group of very similar interventions. Some reviews, however, consider any intervention aimed at achieving a particular outcome. For example, any intervention that aims to improve adherence to tuberculosis treatment (which might include mobile phone messaging, nurse visits or written patient information) or any intervention that aims to improve trust between health care providers and health care recipients (which might include training interventions for health care providers and patient education). For these types of review, it will be necessary to group together similar interventions (e.g., all mobile phone messaging interventions for tuberculosis treatment adherence or all interventions that involve training for health care providers) and to carry out separate iCAT_SR assessments for each of the included intervention groups. It should also be noted that iCAT_SR is not a tool to assess risk of bias or how well an evaluaton study or systematic review has been conducted.

## Discussion

The iCAT_SR version 1 can guide the conduct of systematic reviews of effectiveness by providing review authors with a technique to ‘disaggregate systematically’ [18] and describe key aspects of an intervention. It will allow review authors and others to characterize complex interventions in a uniform framework – a possibility that has been largely lacking to date. The iCAT_SR will also improve understanding of the relationship between intervention components and make more explicit considerations of context and of implementation factors. The tool may also be helpful in planning subgroup analyses to explore effect modification by key differences in intervention complexity – for example, participants exposed to different combinations of intervention components or to interventions targeting different organisational levels. iCAT_SR assessments may also be useful in planning meta-regression to explore the relative importance of different dimensions of intervention complexity in explaining intervention effects, and in conceptualising network meta-analysis to compare the effectiveness of different intervention configurations [29]. All of this may contribute to explaining more clearly which intervention components work and how. Furthermore, by improving the quality of intervention description in systematic reviews, it may also facilitate judgements about applicability of the findings in populations or subgroups [30].

The development of this tool needs to be situated in the evolving science of systematic reviews that proposes the synthesis of multiple sources of data to explain the causal relationship between an intervention and its effects [18, 31]. Other approaches to assessing the complexity of interventions tend to be specific to a clinical or health area [32, 33], and more work is needed to explore how these approaches might be used together with the iCAT_SR. In addition, for behaviour change interventions we need to explore how the iCAT_SR can be used in conjunction with behaviour change intervention frameworks [34] and a taxonomy of behaviour change techniques [35–37]. We also acknowledge that this attempt to assess the degree of complexity of interventions somewhat artificially both splits complexity into a range of dimensions and divides complexity on each dimension into three levels. In practice, all interventions sit on a spectrum from less to more complex for each of the dimensions specified making it difficult to meaningfully talk about a ‘complex intervention’ or an overall degree, or level of, intervention complexity. Nonetheless we see the iCAT_SR as a constructive step in developing a better understanding of the phenomenon of intervention complexity.

The next stage of development will be to further test the iCAT_SR, and the guidance on how to apply it, in selected systematic reviews and also to assess reliability between raters and identify sources of disagreement. We have received offers from a number of review author teams who would like to trial the current version as part of a review of a health intervention, and hope that these pilots will explore both the usability or feasibility of the tool and its usefulness in exploring the nature of the causal pathways for an intervention and assessing the applicability of review findings. The usefulness and usability of the tool for users of systematic reviews also needs to be explored. An iCAT_SR assessment may assist users of reviews in a number of ways: by facilitating clearer description of the intervention that is the focus of the review; by making more explicit aspects of the intervention that may be important for replication in other settings, including the (hypothesized) active components, the behaviours or actions to which the intervention is directed and the level of skill required by those delivering the intervention (dimensions 1, 2 and 5); and by highlighting gaps in knowledge about an intervention, for example regarding the nature of the causal pathway between the intervention and the outcome it is intended to effect (dimension 10) [30]. To aid use of the iCAT_SR, results of assessments could be depicted visually, for example in a chart or pictograph [38], and creating visual presentations will be an important part of future development of the tool. Further work is also needed to evaluate the usefulness to review users of iCAT_SR assessments and different methods of presenting these.

The development of the iCAT_SR has been undertaken by experienced review authors; we also therefore need to assess whether it can be operationalised successfully in the hands of relatively inexperienced review authors as well as the additional workload involved. It is likely that review authors will require some training to use the tool as intended, and there will be a learning curve before the value of the tool becomes apparent. Because judgements are involved in applying iCAT_SR, we recommend that review authors meet at the outset and discuss with some examples how they plan to make their judgements. This should promote agreement within a review team, and make clearer what their judgements mean in the context of a particular review. In addition, we are not advocating that the tool should be used in all reviews of interventions that are viewed as more complex. Rather, the iCAT_SR should be considered in reviews where understanding the complexity of the intervention, and understanding the impacts of this complexity on intervention effects, are important parts of the review question.

The iCAT_SR is not without limitations, the most important of which is likely to be the limited description of intervention components, implementation context and implementation fidelity in primary studies included in a review. Poor reporting is a well acknowledged problem [15, 39] and developing ‘complexity’ extensions to the CONSORT and PRISMA criteria [14] and reporting templates [15] may help to address this through driving up reporting standards. An additional use of the iCAT_SR will be in identifying gaps in reporting and in providing a clear picture of issues to probe when contacting the authors of primary studies.

## Conclusions

The development of the iCAT_SR tool facilitates more in-depth, systematic assessment of the complexity of interventions in systematic reviews of intervention effects and may also contribute to understanding how a complex intervention works. In addition, the tool may have a number of ‘spin off’ effects including improving understanding of the range of different versions of an intervention in the literature; improving intervention description in primary studies; and making it easier for users of reviews to assess the applicability of the intervention to their setting. The iCAT_SR also moves the field away from ‘simplistic’ definitions of what we might view as a complex intervention and suggests that interventions are better described as having degrees of complexity in relation to the iCAT_SR dimensions.

We have added to the title of the tool the suffix ‘version 1’ as we anticipate that reviewers who use the tool will comment and suggest additional modifications. We encourage publication of worked examples of the application of iCAT_SR to different types of interventions (for instance, interventions targeting different governance or financial arrangements for health and social care) from different areas of health and social care (for example, clinical medicine, health systems and public health). We also welcome feedback on the tool and the accompanying guidance (Additional file 4) and plan to review and update these at regular intervals, based on user experience.

## Additional files

Additional file 1: Development of a tool to describe and assess the complexity of interventions in randomised trials (PDF 338 kb)

Additional file 2: iCAT dimensions for assessing the complexity of interventions in the context of randomised trials (PDF 420 kb)

Additional file 3: Dimensions considered and excluded from the iCAT for assessing the complexity of interventions in the context of randomised trials (PDF 241 kb)

Additional file 4: Guidance for using the iCAT_SR tool: intervention Complexity Assessment Tool for Systematic Reviews, version 1.0 (PDF 362 kb)

Additional file 5: Applying the iCAT_SR – example B: systematic review of the effects of interventions aimed at communities to inform and/or educate about early childhood vaccination and qualitative evidence synthesis of parents’ and informal caregivers’ views and experiences of routine early childhood vaccination communication. (PDF 371 kb)

## Abbreviations

CONSORT: Consolidated Standards of Reporting Trials
iCAT: Intervention Complexity Assessment Tool
iCAT_SR: Intervention Complexity Assessment Tool for Systematic Reviews
LHW: Lay health worker
LMICs: Low and middle income countries
MCH: Maternal and child health
MRC: Medical Research Council
PICO: Population, Intervention, Comparison, Outcomes
PRISMA: Preferred Reporting Items for Systematic reviews and Meta-Analyses
