# Supplementary material for: Assessing the complexity of interventions within systematic reviews: development, content and use of a new tool (iCAT_SR)
Source: BMC Med Res Methodol. 2017 Apr 26;17:76. doi: 10.1186/s12874-017-0349-x (PMC5406941; doi:10.1186/s12874-017-0349-x)
Supplement: Supplementary file 1 — Development of a tool to describe and assess the complexity of interventions in randomised trials (PDF 338 kb) [file 12874_2017_349_MOESM1_ESM.pdf]

Additional file 1: Development of a tool to describe and assess the complexity of interventions in randomised trials

The initial version of the iCAT was developed to assess the complexity of interventions being developed for evaluation in randomised trials.

In summary, the development of this tool included the following steps:

- *Developing a list of possible dimensions of intervention complexity:* Two researchers reviewed the available literature on intervention description and complexity and identified potential possible dimensions. An initial list of six dimensions was then compiled, based on those that seemed to best describe intervention complexity and that could be applied pre-trial, to facilitate conceptualisation and piloting of interventions; as part of trial reporting, to improve intervention description; and post-trial, to help assess the replicability of interventions in other settings. Provisional definitions of these dimensions were developed.
- *Discussions and consensus workshops with trialists and implementation researchers:* The proposed dimensions were discussed with a large number of trialists and implementation researchers involved in the Practihc (Pragmatic Randomized Controlled Trials in HealthCare: www.practihc.net) and ReBEQI (Research based Education and Quality Improvement ([1])) projects and other networks. The existing dimensions were then revised and other dimensions suggested.
- *Initial testing of the tool:* Draft versions of the tool were tested against published reports of trial interventions. Some dimensions were adapted or refined if they were difficult to apply. A number of dimensions were excluded from the iCAT, mainly because they were considered to be difficult to assess pre-trial (for example, during intervention development work); they were already covered by an included dimension; they were thought too difficult to assess objectively; or they did not to relate directly to the complexity of an intervention. A list of these excluded dimensions was retained for future consideration (Additional file 3)[1]. A second version of the tool was then developed, along with guidance for its application.
- *Further testing of the tool:* The second version of the tool was assessed for applicability by six researchers, who attempted to apply it to a sample of reports of healthcare and health systems trials. Further adaptation of the guidance for use was then made.

The principal changes between the original version of the iCAT tool developed for assessing complexity in interventions for use in primary studies and the version modified for use in systematic reviews (iCAT_SR) are:

- Four of the ten previously excluded dimensions are now included as “optional”. Although these dimensions may not be applicable in all intervention reviews, they may be useful in some.
- The three level rating system (simple, intermediate, complex) used in the original version was dropped as it was felt that specific descriptors for each dimension would be more helpful, both to those applying the tool and those utilising the results, than a system that could be seen as a “complexity score” (see Additional file 4).

[1] Some of these additional dimensions were subsequently included in the iCAT_SR.

- The guidance on use of the tool was revised to include more detail and advice on use in systematic reviews.
- Changes were made to the layout and appearance of the guidance to improve readability.

References

1. Akl EA, Treweek S, Foy R, Francis J, Oxman AD, ReBeqi group: **NorthStar, a support tool for the design and evaluation of quality improvement interventions in healthcare**. *Implement Sci* 2007, **2**:19.
