# Supplementary material for: Assessing the complexity of interventions within systematic reviews: development, content and use of a new tool (iCAT_SR)
Source: BMC Med Res Methodol. 2017 Apr 26;17:76. doi: 10.1186/s12874-017-0349-x (PMC5406941; doi:10.1186/s12874-017-0349-x)
Supplement: Supplementary file 2 — iCAT dimensions for assessing the complexity of interventions in the context of randomised trials (PDF 420 kb) [file 12874_2017_349_MOESM2_ESM.pdf]

Additional file 2: iCAT dimensions for assessing the complexity of interventions in the context of randomised trials [1]

Dimension	Explanation
Number of discrete, active components included in the intervention compared with the control	This refers to elements of an intervention that are both separate from each other and may, separately, have an effect on outcomes. Components could range from one – for example, a single, one-dose drug intervention – to many components. The latter are sometimes described as multifaceted interventions.
Number of behaviours or actions of intervention recipients to which the intervention is directed	More simple interventions target a single behaviour or action, such as an intervention to encourage physicians to measure blood pressure only for the primary prevention of hypertension, while more complex interventions are directed at multiple changes or barriers example of 'more complex:, an intervention to encourage physicians to implement multiple primary prevention activities such as measuring bloodpressure and blood lipids; screening for colorectal and other cancers; and giving advice about smoking, diet, exercise, alcohol etc.
Number of organisational levels targeted by the intervention	This refers to whether the intervention is directed towards one or more of the following levels: individuals, groups / teams of individuals, or systems. The individual level may include individual consumers, health care providers or health care planners; the group level may include groups of consumers, providers or planners; and the system level may include sections of the health system or geographic or virtual communities. The complexity of an intervention may increase as it targets more levels.
The degree of flexibility or tailoring permitted across sites in intervention implementation / application	Where the implementation of an intervention can be standardised more easily across multiple sites, this may indicate a more simple intervention. In contrast, interventions that are more likely to be modified by the context of intervention delivery, due to differences in the implementation settings, recipients and timing, may be seen as more complex. These interventions may be designed purposively to tailor to individuals or to specific implementation settings, while adhering to a particular set of theoretical or operational principles [2].
The level of skill required by those delivering the intervention	Skill is defined here as the ability to do something arising from training, practice or experience. The level of skill or competence required to deliver an intervention increases as the complexity of the intervention increases. For example, the level of skill required by health care providers to recommend the patient who smokes to 'stop smoking' is less than that required to deliver a multi-session, participatory educational programme to smokers.
The level of skills required by those receiving the intervention in order to meet the intervention's objectives	Those receiving an intervention, such as a taking a single dose drug, require little skill to meet the intervention's objectives (i.e. taking the drug) and such interventions could be seen as simple. In contrast, those receiving an intervention such as an educational programme may need considerable skills, based on training, experience or practice, to interpret the information provided and then to apply it in their setting. Skills may include a high level of health literacy and / or the ability to undertake a specific task, such as monitoring their own blood pressure. Such an intervention could be seen as more complex.

References

1. Lewin S, Oxman A, Glenton C: **Assessing healthcare interventions along the complex-simple continuum: a proposal. Abstract P100**. *14th Cochrane Colloquium.* Dublin, Ireland; October 2006.
2. Hawe P, Shiell A, Riley T: **Complex interventions: how "out of control" can a randomised controlled trial be?** *Bmj* 2004, **328**(7455):1561-1563.
