# Supplementary material for: Assessing the complexity of interventions within systematic reviews: development, content and use of a new tool (iCAT_SR)
Source: BMC Med Res Methodol. 2017 Apr 26;17:76. doi: 10.1186/s12874-017-0349-x (PMC5406941; doi:10.1186/s12874-017-0349-x)
Supplement: Supplementary file 3 — Dimensions considered and excluded from the iCAT for assessing the complexity of interventions in the context of randomised trials (PDF 241 kb) [file 12874_2017_349_MOESM3_ESM.pdf]

**Additional file 3: Dimensions considered and excluded from the iCAT for assessing the complexity of interventions in the context of randomised trials**

| Dimension | Reasons for exclusion |
|---|---|
| 1. Differential effects on recipient groups, including patients and practitioners, and across different organisational and systems settings | • Difficult to assess pre-trial<br>• There may be insufficient data to assess differential effects post trial |
| 2. The length of the 'causal pathway' between the intervention and the outcome it is intended to effect | • The length of causal pathway between an intervention and an outcome depends on the outcome being measured, i.e. it is not a necessarily a property of the intervention itself<br>• There is often little empirical evidence on which to construct causal pathways<br>• The number of steps within the pathway depends on how this is envisaged |
| 3. The extent of interaction between intervention components / the independence of intervention components | • Dimension exploring the number of discrete, active components included within the intervention covers similar territory<br>• Assessment of the extent of interaction / independence may be very subjective |
| 4. Predictability of intervention effects / uncertainty regarding the effects of the intervention | • Assessment likely to be very subjective<br>• May reflect poor state of knowledge rather being inherent to complex interventions |
| 5. Differential uptake / exposure to the intervention across different recipient groups or settings | • Replaced by the degree of site-to-site variation in intervention implementation / application |
| 6. The degree to which the effects of the intervention are dependent on the context or setting in which it is implemented (e.g. would the same intervention have the same effects in primary care clinics and tertiary level hospitals, or in one country compared to another?) | • Difficult to assess pre-trial<br>• There may be insufficient data to assess differential effects post trial, particularly since many interventions are implemented in one setting or context only |
| 7. The extent to which the 'active ingredient' of the intervention can be specified | • Likely to be highly subjective, given the current state of knowledge<br>• To some extent captured by the following dimension: the number of discrete, active components included within the intervention |
| 8. The ways in which the effectiveness of an intervention is modified by patient, provider and health care delivery factors | • Covered by the dimensions: (1) the level of skill required by those delivering the intervention; (2) the level of skill required by those receiving the intervention; and (3) the |

| | |
|---|---|
| | degree of site-to-site variation in intervention application. |
| 9. The infrastructure needed to deliver the intervention | • Not a characteristic of the intervention itself<br>• Difficult to assess from published reports |
| 10. Predictability or measurability of effect modifiers | • Concerns may reflect poor state of knowledge rather than an underlying relationship between the complexity of an intervention and the difficulty in measuring effect modifiers |
