# Supplementary material for: Assessing the complexity of interventions within systematic reviews: development, content and use of a new tool (iCAT_SR)
Source: BMC Med Res Methodol. 2017 Apr 26;17:76. doi: 10.1186/s12874-017-0349-x (PMC5406941; doi:10.1186/s12874-017-0349-x)
Supplement: Supplementary file 4 — Guidance for using the iCAT_SR tool: intervention Complexity Assessment Tool for Systematic Reviews, version 1.0 (PDF 362 kb) [file 12874_2017_349_MOESM4_ESM.pdf]

Cochrane Methods

Guidance for using the iCAT_SR: Intervention Complexity Assessment Tool for Systematic Reviews

Version 1.0

Simon Lewin
Maggie Hendry
Jackie Chandler
Andrew D Oxman
Susan Michie
Sasha Shepperd
Barnaby C Reeves
Peter Tugwell
Karin Hannes
Eva A Rehfuess
Vivien Welch
Joanne E Mckenzie
Belinda Burford
Jennifer Petkovic
Laurie M Anderson
Janet Harris
Jane Noyes

Trusted evidence.
Informed decisions.
Better health.

Author affiliations:

Simon Lewin[1, 2], Maggie Hendry[3], Jackie Chandler[4], Andrew D Oxman[1], Susan Michie[5], Sasha Shepperd[6], Barnaby C Reeves[7], Peter Tugwell[8], Karin Hannes[9], Eva A Rehfuess[10], Vivien Welch[11, 12, 13], Joanne E Mckenzie[14], Belinda Burford[15], Jennifer Petkovic[16], Laurie M Anderson[17], Janet Harris[18], Jane Noyes[19]

[1] Norwegian Institute of Public Health, Oslo, Norway
[2] Health Systems Research Unit, South African Medical Research Council, Cape Town, South Africa
[3] North Wales Centre for Primary Care Research, Bangor University, Bangor, United Kingdom
[4] Cochrane, Cochrane Central Executive, London, United Kingdom
[5] Centre for Behaviour Change, University College London, London, United Kingdom
[6] Nuffield Department of Population Health, University of Oxford, Oxford, United Kingdom
[7] Clinical Trials and Evaluation Unit, School of Clinical Sciences, University of Bristol, Bristol, United Kingdom
[8] Institute of Population Health, University of Ottawa, Ottawa, Canada
[9] Social Research Methodology Group, Centre for Sociological Research, Faculty of Social Sciences, KU Leuven, Leuven, Belgium
[10] Institute for Medical Informatics, Biometry and Epidemiology, University of Munich, Munich, Germany
[11] Bruyère Research Institute, Bruyère Continuing Care, Ottawa, Ontario, Canada
[12] School of Epidemiology, Public Health and Preventive Medicine, University of Ottawa, Ottawa, Canada
[13] Ottawa Hospital Research Institute, Ottawa, Ontario, Canada
[14] School of Public Health and Preventive Medicine, The Alfred Centre, Monash University, Melbourne, Victoria, Australia
[15] Cochrane Public Health Group and Jack Brockhoff Child Health and Wellbeing Program, Melbourne School of Population and Global Health, University of Melbourne, Melbourne, Victoria, Australia
[16] Campbell and Cochrane Equity Methods Group, Centre for Global Health, Institute of Population Health, University of Ottawa, Ottawa, Canada
[17] Department of Epidemiology, School of Public Health, University of Washington, Seattle, USA
[18] School of Health and Related Research (ScHARR), University of Sheffield, Sheffield, United Kingdom
[19] School of Social Sciences, Bangor University, Bangor, United Kingdom

# Corresponding author:

Simon Lewin: simon.lewin@fhi.no

Citation:

Lewin S et al. Guidance for using the iCAT_SR: Intervention Complexity Assessment Tool for Systematic Reviews, Version 1.0. Cochrane Methods; 2016. Available at [*weblink to be inserted after publication*]

Date:

23 December 2016

Trusted evidence.
Informed decisions.
Better health.

Guidance for using the iCAT_SR: Intervention Complexity Assessment Tool for Systematic Reviews, version 1.0

Background:

This tool has been developed to facilitate a more systematic and transparent approach to assessing where a health intervention lies on the spectrum from more simple to more complex in the context of systematic reviews. The tool is an aid to disaggregating intervention 'components' and their delivery, and assessing interventions across a set of 'dimensions' that categorise levels of intervention complexity [1, 2].

Undertaking this 'complexity assessment' may assist review authors in:

- Systematically and consistently describing and disaggregating interventions (and comparisons) in terms of their component parts. This will assist in formulating the PICO review question, developing criteria for including studies and selecting studies for inclusion
- Developing search strategies for a review
- Extracting data
- Developing hypotheses regarding causal pathways, developing logic models [3, 4] and constructing graphical representations of interventions [5]
- Analysing data and undertaking meta-analyses, including classifying or grouping interventions according to which components they include; identifying explanatory factors that may explain differences in results across studies and across subgroups within studies; and interpreting review findings
- Making judgements about the applicability of review findings in populations or subgroups [6], by improving the quality and usefulness of intervention descriptions
- Presenting review findings

The tool comprises ten dimensions to assess intervention complexity (Tables 1 and 2). Dimensions 1 – 6 may be considered "core" dimensions. Dimensions 7 – 10 are optional; they may not be useful for all interventions (and therefore reviews) and may be more difficult to apply, in part because the information needed to make an assessment may often be poorly reported or absent. Review authors may also identify additional dimensions, not described here, that are

applicable to particular intervention(s) included in a review; and these may be added to an assessment.

The tool may facilitate identification of those components of an intervention that are intended to be 'stable' and those that are expected to be tailored or specific to contextual or individual factors. Components that are stable can be viewed as "structural" while those that are expected to be tailored can be viewed as "functional" (and may vary according to time, place and context). Dimension 4 of this tool focuses on the extent to which an intervention (or its components) is tailored or flexible. Dimensions 7, 8 and 9 are aligned towards assessment of the interactions between intervention components and between the effects of an intervention and context, recipient and provider factors.

Using the tool:

Before undertaking a complexity assessment using the iCAT_SR, it may be helpful to describe the following in relation to your review:

- The intervention/s and the comparison (including usual care)
- Who delivered the intervention
- To whom the intervention was directed (which may include individuals, groups of individuals and other entities)
- Whose behaviour / action the intervention intended to change (where applicable).

For each iCAT_SR dimension, an intervention can be graded on one of three levels, ranging from more simple to more complex (see Tables 1 and 2). . For some components, it is also possible to select 'varies' where that particular component varies across interventions to be considered for the review or 'unclear or unable to assess' when the information needed to make an assessment is not available.

The assessment for each iCAT_SR component should be accompanied by a brief summary of the information underpinning the assessment (the support for judgement), so as to improve transparency and help readers understand the judgements made. A 'dummy table' for these assessments is included below (Table 3). Information for the support for judgement may be drawn from multiple sources: the published study report; ancillary papers on the study, including qualitative process evaluations; and information obtained from study authors. Where a review of effectiveness has a linked qualitative evidence synthesis that explores how the intervention works and factors affecting its implementation, this additional information may be very helpful in making assessments for the complexity dimensions. Review authors are encouraged to include verbatim quotes from study reports or author correspondence in their support for judgement.

At the development and protocol stages of a review, review authors may not have sufficient information to make definitive judgements regarding all of the ten dimensions. In these cases, we recommend that the review team make a provisional judgement or develop an *a priori*, clear hypothesis and then note these hypotheses in their support for judgement. Information emerging at the analysis stage, such as the results of subgroup analyses or meta-regresssion, may allow judgements to be made with greater confidence. The iCAT_SR assessment can then be amended.

We acknowledge that some of the examples given in this guidance are oriented towards health system and public health interventions and may need to be interpreted / adapted for review authors working in other content areas.

Further information on the tool:

Further information on the tool, including how it was developed and worked examples, is available in this paper [2].

Table 1: Core dimensions of the iCAT_SR

Dimension 1<br>***Active components[1] included in the intervention, in relation to the comparison[2]***		Elaboration / explanation
		1. An intervention component is defined as a discrete, active element of the intervention that could be implemented independently of other elements, e.g. an educational booklet for consumers; clinical guidelines for practitioners; a single drug intervention; a discrete surgical procedure. Activities that are clearly stated as being part of data collection rather than the intervention should not be counted as an intervention component. For example, where participants are asked to keep a log of events, such as falls or home visits.<br>2. Identification of the number of components included in the intervention needs to be undertaken in relation to the comparison intervention. For example, in a review comparing laparoscopic surgery with open surgery for colon cancer, the number of components in the intervention should be assessed as one (the surgical procedure). All other procedures (pre-op, anaesthesia, post-op care) would be the same in both intervention and comparison and are therefore not part of the assessed intervention. In contrast, in a review comparing prostate surgery with 'watchful waiting' for prostate enlargement, the number of components in the intervention should be assessed as 'three or more' (pre-op, anaesthesia, surgery, post-op care etc.). If one component of the intervention is made up of multiple, potentially independent sub-components, each of these should be counted as a separate intervention component. For example, a home 'falls prevention' intervention might include a medical assessment, an environmental assessment, changes to the home environment and referral of problems to other health and social services.
**More than one component *and* delivered as a bundle**	The intervention includes more than one component and some or all of these components need to be delivered as a bundle.[3]	3. A bundle is a set of intervention components that are intended to be used together to improve patient outcomes. It is expected that all of the elements of the bundle must be performed in a series of steps by one healthcare team (or other entity) within a particular timeframe for the intervention to impact on the designated outcomes. Changing a step in the process may alter the intended effect. For example, the ventilator bundle, developed to prevent ventilator-associated complications, comprises four core components: administering deep vein prophylaxis; administering medications to prevent gastric ulceration; elevating the head of the bed between 30 and 45 degrees; and providing a daily break in sedation for the purpose of assessing whether the patient can breathe independently of the ventilator.
**More than one component**	The intervention includes more than one component. These components may be integrated into package.[4]	4. An integrated package is a group of intervention components that are intended to be used together but do not necessarily need to be performed in a specific order or timeframe, or simultaneously, to impact on the designated outcomes. For instance, intervention components may target different members of multi- professional teams or different levels of care such as primary, secondary and tertiary care. An integrated package could bring together different facets of care such as inputs, delivery, management and organization of services related to diagnosis, treatment, care, rehabilitation and health promotion; it could involve the integration of health services with social care, housing, education etc. For example, the NICE quality standard on drug use disorders [7] states that people accessing drug treatment should be offered a comprehensive assessment of their drug use and resources for recovery; an assessment of their personal, social and mental health needs in relation to family and carers; and support to access services which promote recovery and reintegration including housing, education, employment, personal finance, healthcare and mutual aid.
**One component**	The intervention includes one component only.	
**Varies**	Varies across interventions to be considered for / included in the review.<br>*(Note that if this category is selected, review authors should consider whether the interventions included in the review are as similar as originally thought and whether this has implications for the review's inclusion criteria.)*	

Dimension 2

Behaviours[5] or actions of intervention recipients or participants to which the intervention is directed[6]

**Multi-target**	Intervention directed at three or more behaviours or actions.
**Dual target**	Intervention directed at two behaviours or actions.[7]
**Single target**	Intervention directed at one behaviour or action only.[8]
**Varies**	Varies across interventions to be considered for / included in the review. *(Note that if this category is selected, review authors should consider whether the interventions included in the review are as similar as originally thought and whether this has implications for the review's inclusion criteria.)*

Elaboration / explanation

5. Behaviours or actions include taking a medication, changing a particular practice, improving knowledge or undergoing a surgical procedure. Behaviours or actions are targeted by the active components of the intervention. Please note the following: (a) a behaviour may include not doing something, such as not smoking; (b) for some interventions, the intervention recipients may not undertake any behaviours or actions in the context of the intervention, e.g. in a surgical intervention, where the actions are undertaken by the surgical team rather than the intervention recipients. These interventions should be assessed as 'not applicable' for this dimension.
6. Assessment of the number of behaviours or actions to which the intervention is directed is not dependent on, or necessarily related to, the outcome/s measured by the trials included in a review. For example, an intervention directed at physicians to improve their management of cardiac risk factors in patients could be assessed by several outcomes: knowledge of cardiac risk factor management strategies; implementation of these strategies; prescribing patterns; changes in the health behaviours of patients; and changes in health outcomes, such as mortality from cardiac events. The outcome(s) chosen do not impact on the behaviours or actions (in this case, physicians' management of cardiac risk factors) to which the intervention is directed. Another example is an intervention directed at increasing exercise and walking among elderly people where the measured outcome is the number of falls in the home. Here again the behaviours targeted are not the same as the outcomes measured.
7. If the intervention is directed at a set of linked behaviours, e.g. the implementation of a guideline for a range of actions (such as asthma medication, education and monitoring); self- monitoring; or fall prevention in the elderly, then it should be graded as multi-target (linked) or dual-target (linked).
8. A single dose single drug intervention is targeted at one action – taking a medication – and would therefore be scored as single target. In contrast, chronic medication involves prolonged repetition of a single action and should therefore be scored as multi target (linked). Other examples of a single behaviour or action include interventions to promote hand-washing; to install fire alarms; and to use safety belts in cars.

Dimension 3

***Organisational levels and categories targeted by the intervention*[9]**

**Multi-level**	Intervention directed at two or more levels.
**Multi-category**	Intervention directed at two or more categories of individuals within the individual level (e.g. primary care professionals and primary care patients).
**Single category**	Intervention directed only at single category of individuals within the individual level (e.g. professionals *or* patients *or* policy makers).[10]

Elaboration / explanation

9. Level refers to whether the intervention was directed at one of more of the following: individuals (consumers, professionals, policy makers); groups or teams of individuals (staff of clinics, patient support group etc.); or systems (communities, health systems, organisations (such as hospitals), policy networks). Categories are groups, such as nurses or patients, within those levels. A judgement about the number of levels targeted by the intervention needs to take into account the focus of the research question.
   - Example 1: consider a new drug compared with placebo. Although patients are the ultimate recipients of the intervention, the physicians themselves also receive some form of intervention since some change to their standard practice may need to be introduced (e.g. they may be prescribing for a condition they never prescribed for before). In this case, since the research question is concerned with the effectiveness of drug A compared with placebo, and all physicians will receive the intervention to change their standard practice, the relevant intervention should be considered to be directed only at a single category of individuals – the patients – and should therefore be graded as 'single category' (We are grateful to Kevin Thorpe for this example).
   - Example 2: if an intervention is mediated through, say, nurses (who require training to deliver the intervention) but is directed at patients, then the number of organisational levels or categories targeted should be graded as 'single category' as the patients are the target of the intervention.
   - Example 3: if an intervention is directed at one category of individuals (e.g., patients), but groups of patients are randomised, then the intervention should still be graded as 'single category' as it is targeted at a single category of individuals only.
10. For cluster randomized trials, consider the levels targeted by the intervention. If the intervention is directed towards groups of clinicians *and* individual patients, it should be assessed as 'multi- category'. If the intervention is directed towards groups of clinicians *or* groups of patients, it should be assessed as 'single category' as this constitutes only one category of individuals (see footnote above). If the intervention is directed at some of the individuals within a cluster rather than at the cluster as a whole, e.g. an intervention directed at a physicians within a multi-professional primary care centre rather than at all the health care professionals in the centre, then the intervention should be assessed as 'single category'.

Dimension 4

The degree of tailoring intended or flexibility permitted across sites or individuals in applying or implementing the intervention[11]

**Highly tailored / flexible**	High degree of variation in implementation from site to site permitted *and / or* intervention designed to tailor to individuals or specific implementation settings (i.e. the protocol or instructions for applying the intervention are highly flexible, allowing implementers considerable leeway in deciding how to formulate and apply it [8]).
**Moderately tailored / flexible**	Some variation in implementation from site to site permitted (i.e. some components of the intervention are tailored / flexible while others are not).[12]
**Inflexible**	Intervention implementation highly standardised with minimal variation from site to site.[13]
**Varies**	Varies across interventions to be considered for / included in the review. *(Note that if this category is selected, review authors should consider whether the interventions included in the review are as similar as originally thought and whether this has implications for the review's inclusion criteria.)*

Elaboration / explanation

11. This dimension should be assessed by looking at the tailoring intended or flexibility permitted for each component of the intervention (see Dimension 1). Tailoring implies that the intervention is intended to be modified for specific individuals, settings or circumstances, whereas flexibility implies leeway for modification *if desired*. Interventions may be modifiable in both content (e.g. variation in the components received by sites or individuals) and form (variation in the ways in which the components are delivered across sites or individuals).
12. For example, an intervention may include tailored counselling for patients, standardised patient materials and a standardised medical assessment. Most forms of counselling and related interventions, such as psychotherapy, are tailored towards individual needs and would therefore be expected to vary across individuals.
13. For example, a standardised, non-tailored reminder letter; the insertion of a cardiac pacemaker following a standard protocol; or a standardised drug regimen.

Dimension 5

The level of skill required by those delivering the intervention in order to meet the intervention objectives[14]

**High level skills**	Extensive specialised skills required[15] i.e., new skills in addition to expected existing skills AND / OR the extension of existing skills to a highly specialised area AND / OR skills requiring extensive additional training.
**Intermediate level skills**	Some specialised skills required, i.e. a small extension to the expected existing skills of professionals, decision makers or consumers. This may facilitate the better performance of tasks that they already undertake and involve a short period of training.
**Basic skills**	No specialised skills required.
**Varies**	Varies across interventions to be considered for / included in the review. *(Note that if this category is selected, review authors should consider whether the interventions included in the review are as similar as originally thought and whether this has implications for the review's inclusion criteria.)*

Elaboration / explanation

14. Again, this should be assessed in relation to the comparison intervention.
15. Skill is defined as the ability to do something, arising from training, practice or experience.

Dimension 6

The level of skill[16] required f~~or the targ~~eted behaviour when entering the included studies by those <u>receiving</u> the intervention (consumers, professionals, planners), in order to meet the intervention objectives[17]

**High level skills**	Extensive specialised skills required.[18]
**Intermediate level skills**	Some specialised skills required.[19]
**Basic skills**	No specialised skills required.[20]
**Varies**	Varies across interventions to be considered for / included in the review. *(Note that if this category is selected, review authors should consider whether the interventions included in the review are as similar as originally thought and whether this has implications for the review's inclusion criteria.)*

Elaboration / explanation

16. Skill is defined as the ability to do something, arising from training, practice or experience.
17. This assessment needs to be made in relation to the review's inclusion criteria.
18. For professionals, this is defined as basic professional training AND additional training of some sort (e.g., as a specialist physician or in the use of a particular procedure or technique).
19. For professionals, this is defined as their basic professional training, e.g. as a physiotherapist or nurse. If the recipient group includes a mix of 'non-specialist' and specialist health care providers (e.g. professional nurses and intensive care nurses) then, by implication, fewer specialist skills are required and the intervention should be scored as 'intermediate'. For patients / consumers, this category should be selected if the inclusion criteria for the review specify that patients / consumers in included studies need specific skills to be eligible for entry (e.g. must be proficient in the use of internet search engines) or if patients / consumers are given specific training prior to the study entry to assist them in performing the targeted behaviour (e.g. training in the use of a particular medical device such as a home blood glucose meter).
20. For patients / consumers, this grade should always be chosen if all comers are enrolled into the included studies. This category should not be used for professionals unless they are not using their professional skills within the context of the intervention. For example, the provision of leaflets to specialist surgeons to encourage them to wash their hands more often on hospital wards should be assessed as 'basic' as hand-washing does not require any specialised or professional skills on the part of surgeons.

Table 2: Optional dimensions of the iCAT_SR

Dimension 7

The degree of interaction between intervention components, including the independence / interdependance[21] of intervention components[22]

**High level interaction**	There is substantial interaction or inter-dependency between intervention components or actions i.e. the delivery of one intervention component impacts on the delivery of another, resulting in a synergistic effect.
**Moderate interaction**	There is some degree of interaction but no evidence of synergistic effects or dysynergistic effects.
**Independent**	The intervention has only one component or action, or the components act independently.
**Varies**	Varies across interventions to be considered for / included in the review. *(Note that if this category is selected, review authors should consider whether the interventions included in the review are as similar as originally thought and whether this has implications for the review's inclusion criteria.)*
**Unclear or unable to assess**	

Elaboration / explanation (adapted from [9, 10])

21. The effectiveness of an intervention may depend on the combination of components delivered and / or the sequence of delivery. Where such interdependencies exist, they can be described as:
    - i. Contemporaneous: The effect of one intervention component depends on another intervention component being present at the same time. On their own, each component may be less effective, ineffective, or harmful.
    - ii. Temporal: The effect of one intervention component depends on another component being present beforehand. On their own, each component may be less effective, ineffective, or harmful.
22. Consider whether the components are only hypothesized to work, or to work better, as a package? Are there synergistic ("added value") or dysynergistic effects?
    - i. Synergistic: Intervention components interact in ways that the total effect is greater than the sum of the individual effects of the components.
    - ii. Dysynergistic: Intervention components act in ways that the total effect is less than the sum of the individual effects of the components.

Dimension 8

The degree to which the effects of the intervention are dependent on the context or setting in which it is implemented[23]

**Highly context dependent**	The effects of the intervention are likely to be strongly dependent on the implementation setting.
**Moderately context dependent**	The effects of the intervention are likely to be transferrable across a limited range of settings only (e.g. only within a specific country or health system).
**Independent of context**	The effects of the intervention do not appear to be strongly dependent on the implementation setting, i.e. it is anticipated that the effects of the intervention will be similar across a wide range of contexts or settings.
**Varies**	Varies across interventions to be considered for / included in the review. *(Note that if this category is selected, review authors should consider whether the interventions included in the review are as similar as originally thought and whether this has implications for the review's inclusion criteria.)*
**Unclear or unable to assess**	

Elaboration / explanation

23. Effects may be dependent on interactions between the intervention and societal, political, economic, health system or environmental context. For example, an intervention may not have the same effects in primary care clinics and tertiary level hospitals, or in a health system in which care is free at the point of contact compared to one in which that is not the case. Other considerations may include socioeconomic factors, including income, education and literacy levels.

Dimension 9

The degree to which the effects of the intervention are changed by recipient[24] or provider[25] factors

**Highly dependent on individual-level factors**	The effects of the intervention are modified by both recipient and provider factors.
**Moderately dependent on individual-level factors**	The effects of the intervention are modified by one of recipient or provider factors.
**Largely independent of individual-level factors**	The effects of the intervention are not modified substantially by recipient or provider factors.
**Varies**	Varies across interventions to be considered for / included in the review. *(Note that if this category is selected, review authors should consider whether the interventions included in the review are as similar as originally thought and whether this has implications for the review's inclusion criteria.)*
**Unclear or unable to assess**	

Elaboration / explanation

24. For example, the effect of an intervention to promote behaviour change (smoking, diet, exercise) could be modified by recipients' age, readiness to change, self-efficacy, peer support etc.
25. For example, therapist-dependent interventions, such as counselling, where the intervention is a combination of the therapist effect and the therapy or procedure and the effectiveness is potentially dependent on both.

Dimension 10

The nature of the causal pathway[27] between the intervention and the outcome it is intended to effect

**Pathway variable, long**	The causal pathway includes three or more steps between intervention and outcome or occurs over a long time period; is not linear, or is variable; and / or more than one causal pathway has been proposed.
**Pathway linear, long**	The causal pathway is linear but there are three or more steps between intervention and outcome.
**Pathway linear, short**	The causal pathway is clear, short (only one or two steps), direct, linear.
**Varies**	Varies across interventions to be considered for / included in the review. *(Note that if this category is selected, review authors should consider whether the interventions included in the review are as similar as originally thought and whether this has implications for the review's inclusion criteria.)*
**Unclear or unable to assess**	

Elaboration / explanation

27. We are referring here to pathways that involve human actions (such as behaviours) or actions within organisations or systems rather than biological pathways. While biological pathways may also be long and non- linear, they are not the focus of this dimension.

To map the causal pathway, users should describe (or hypothesize if the pathway is unclear) how the intervention is intended to work. The (hypothesised) causal pathway may be described explicitly in included studies but is often implicit or unclear. Pathways may be described narratively or displayed graphically in different ways such as logic models, charts or tables so as to show the important elements and relationships within the causal pathway [3]. Displaying the hypothesized causal pathway graphically may be useful for both developing the pathway and, later, for review users. Recent papers provide guidance on developing and using logic models in the context of systematic reviews [3, 4].

Table 3: iCAT_SR assessment reporting table

Core dimension	Description of the intervention in the review	Judgement	Support for judgement
1. Active components included in the intervention, in relation to the comparison			
2. Behaviour or actions of intervention recipients or participants to which the intervention is directed			
3. Organisational levels and categories targeted by the intervention			
4. The degree of tailoring intended or flexibility permitted across sites or individuals in applying or implementing the intervention			
5. The level of skill required by those delivering the intervention in order to meet the intervention's objectives			
6. The level of skill required for the targeted behaviour when entering the included studies by those receiving the intervention, in order to meet the intervention's objectives			
**Optional dimension**	**Description of the intervention in the review**	**Judgement**	**Support for judgement**
7. The degree of interaction between intervention components, including the independence / interdependence of intervention components			
8. The degree to which the effects of the intervention are dependent on the context or setting in which it is implemented			
9. The degree to which the effects of the intervention are modified by recipient or provider factors			
10. The nature of the causal pathway between the intervention and the outcome it is intended to effect			

References

1. Lewin S, Oxman A, Glenton C: **Assessing healthcare interventions along the complex-simple continuum: a proposal. Abstract P100**. *14th Cochrane Colloquium.* Dublin, Ireland; October 2006.
2. Lewin S, Hendry M, Chandler J, Oxman A, Michie S, Shepperd S, Reeves B, Tugwell P, Hannes K, Rehfuess E *et al*: **Assessing the complexity of interventions within systematic reviews: development, content and use of a new tool (iCAT_SR)**. *Implementation Science* 2017, **Submitted**.
3. Anderson LM, Petticrew M, Rehfuess E, Armstrong R, Ueffing E, Baker P, Francis D, Tugwell P: **Using logic models to capture complexity in systematic reviews**. *Res Synth Methods* 2011, **2**(1):33-42.
4. Kneale D, Thomas J, Harris K: **Developing and Optimising the Use of Logic Models in Systematic Reviews: Exploring Practice and Good Practice in the Use of Programme Theory in Reviews**. *PLoS One* 2015, **10**(11):e0142187.
5. Perera R, Heneghan C, Yudkin P: **Graphical method for depicting randomised trials of complex interventions**. *BMJ* 2007, **334**(7585):127-129.
6. Burford B, Lewin S, Welch V, Rehfuess E, Waters E: **Assessing the applicability of findings in systematic reviews of complex interventions can enhance the utility of reviews for decision making**. *Journal of clinical epidemiology* 2013, **66**(11):1251-1261.
7. NICE: **Drug use disorders in adults**. In. London, UK: National Institute for Health and Care Excellence. Available at: nice.org.uk/guidance/qs23; 2012.
8. Thorpe KE, Zwarenstein M, Oxman AD, Treweek S, Furberg CD, Altman DG, Tunis S, Bergel E, Harvey I, Magid DJ *et al*: **A pragmatic-explanatory continuum indicator summary (PRECIS): a tool to help trial designers**. *CMAJ* 2009, **180**(10):E47-57.
9. Grimshaw JM, Freemantle N, Langhorne P, Song F: **Complexity and systematic reviews: report to US Congress Office of Technology Assessment**. In. Washington DC, USA: Office of Technology Assessment; 1995.
10. Squires JE, Valentine JC, Grimshaw JM: **Systematic reviews of complex interventions: framing the review question**. *Journal of clinical epidemiology* 2013, **66**(11):1215-1222.
