# Supplementary material for: Assessing the complexity of interventions within systematic reviews: development, content and use of a new tool (iCAT_SR)
Source: BMC Med Res Methodol. 2017 Apr 26;17:76. doi: 10.1186/s12874-017-0349-x (PMC5406941; doi:10.1186/s12874-017-0349-x)
Supplement: Supplementary file 5 — Applying the iCAT_SR – example B: systematic review of the effects of interventions aimed at communities to inform and/or educate about early childhood vaccination and qualitative evidence synthesis of parents’ and informal caregivers’ views and experiences of routine early childhood vaccination communication. (PDF 371 kb) [file 12874_2017_349_MOESM5_ESM.pdf]

Additional file 5: Applying the iCAT_SR – example B: systematic review of the effects of interventions aimed at communities to inform and/or educate about early childhood vaccination [1] and qualitative evidence synthesis of parents' and informal caregivers' views and experiences of routine early childhood vaccination communication [2]

Core dimension	Description of the intervention in the review	Judgement	Support for judgement
**1. Active components included in the intervention, in relation to the comparison**	'We included interventions aimed at communities, with a broad audience and purpose…and that were intended to inform and/or educate about vaccination in children six years and younger.' [1]	*Varies*	The active component is a communication intervention aimed at communities. Some eligible interventions include only one component (e.g., an information pamphlet) while other include more than one component (e.g., web-based programmes that may include information provision, discussion of this information and links to other relevant material).
**2. Behaviour or actions of intervention recipients or participants to which the intervention is directed**	'The interventions aim to increase participants' levels of knowledge and/or change their attitudes regarding vaccination. Changes in knowledge and/or attitudes can be regarded as intermediate outcomes, and may lead to at least two more distal outcomes: (1) a change in the number of participants who make informed decisions regarding childhood vaccination (which may include the decision not to vaccinate); and (2) a change in childhood vaccination rates.' [1]<br><br>Primary review outcomes: (1) 'Immunisation status of child (e.g. immunisation status up-to-date as defined by the author of the included study: receipt of one or more vaccines). (2) Any other measures of vaccination status in children (e.g. immunisation status for a specific vaccine, number of vaccine doses received).' [1]	*Multi-target*	The interventions are directed at what could be considered a linked set of behaviours that may ultimately impact on people's decisions regarding the vaccination of their child and on vaccination uptake. These behaviours may include considering the information provided, discussing this information with relevant stakeholders in the household, and following through on the decision made.
**3. Organisational levels and categories**	'We included interventions aimed at communities, with a broad audience and purpose…and that were intended to inform and/or educate about vaccination in children six years	*Multi-level*	The intervention is directed at two levels: communities as a whole and specific groups

**targeted by the intervention**	and younger….We defined interventions aimed at communities as those directed at a geographic area and/or interventions directed to groups of people who share at least one common social or cultural characteristic.' [1]<br>'We included interventions which targeted groups of people (the general public), including, for example, parents and other caregivers and family members of young children, community leaders, teachers, health personnel (as part of a wider community intervention) and other influential community members.' [1]		within communities, including parents and caregivers, community leaders and teachers.
**4. The degree of tailoring intended or flexibility permitted across sites or individuals in applying or implementing the intervention**	'We included interventions aimed at communities, with a broad audience and purpose…and that were intended to inform and/or educate about vaccination in children six years and younger.' [1]<br><br>'The interventions may include: printed materials such as brochures, pamphlets, posters or fact sheets; electronic media such as videos, slide shows, web-based programmes or audio recordings; and large-scale media such as billboards, newspaper, television and radio.' [1]	*Varies*	Because the review included any intervention aimed at communities and intended to inform and/or educate about childhood vaccination, the range of intervention is wide. Some interventions could be implemented in a highly standardised way (e.g., a television advert) while others might allow variation from site to site (e.g., facilitated community discussions on the pros and cons of vaccination and ways to improve vaccination in a particular setting [3]).
**5. The level of skill required by those delivering the intervention in order to meet the intervention objectives**	'Delivery mechanisms may include: printed materials such as brochures, pamphlets, posters or fact sheets; electronic media such as videos, slide shows, web-based programmes, virtual online communities or audio recordings; large-scale media such as billboards, newspaper, television and radio; and face-to-face communication with groups of people.' [1]	*Varies*	Some community-aimed interventions to inform or educate may require no specialised skills among those delivering them (e.g., distributing pamphlets) while others may require extensive specialised skills (e.g., facilitating discussion groups in communities [3]).

**6. The level of skill required for the targeted behaviour when entering the included studies by those receiving the intervention, in order to meet the intervention objectives**	‘We included interventions which targeted groups of people (the general public)…Some of these groups are the 'end' target group for vaccination communication interventions (such as parents and other caregivers) while other groups are 'intermediaries' who are targeted because of their ability to convey information to or educate the end target group.’ [1]	*Basic skills*	No specialised skills were required of the people participating in the studies.
**Optional dimension**	**Description of the intervention in the review**	**Judgement**	**Support for judgement**
**7. The degree of interaction between intervention components, including the independence / interdependence of intervention components**	The degree of interaction between intervention components was not specified in the review inclusion criteria, described explicitly in the data extraction or analysed as part of the review.	*Unclear or unable to assess*	Not described or analysed in the review. Likely to vary across the included studies. For example, interventions such as facilitated community discussions may have temporal interdependency as the ability to deliver information to discussion groups depends on establishing functioning groups in the first instance.

**8. The degree to which the effects of the intervention are dependent on the context or setting in which it is implemented**	Both of the studies included in the effectiveness review were conducted in community settings in low and middle income countries. This review concluded that 'The two studies identified were conducted in quite similar settings, therefore it is difficult to assess the transferability of the review findings.' [1]<br><br>The studies included in the qualitative evidence synthesis were drawn from a wide range of low, middle and high income countries and the synthesis noted that context was generally poorly described in many of the included studies. However, a number of the synthesis findings suggest that effects are likely to be dependent on context for many relevant interventions. For example, parents found it difficult to remember information given during vaccination appointments as they were distracted and worried about their child and also wanted information to be available in a wider range of contexts, including in parents' groups and online forums [2].	*Unclear or unable to assess*	The available data make it difficult to assess this dimension reliably. Updates of these reviews may provide further information.
**9. The degree to which the effects of the intervention are changed by recipient or provider factors**	This aspect was not considered in detail in the effectiveness review. The qualitative synthesis, however, highlights the important of both recipient and provider factors. For example, some parents questioned the objectivity of the health care providers involved in vaccination communication and parents' attitudes towards vaccination also influenced the vaccination information sources that they trusted [2].	*Highly dependent on individual-level factors*	Most interventions to inform and educate about childhood vaccination are intended to change the attitudes of recipients and influence their intentions and behaviours in relation to vaccination uptake. We would therefore expect these interventions to be dependent on recipients' trust of information sources such as health care providers as well as their readiness for behaviour change, their self-efficacy etc.
**10. The nature of the causal pathway between the intervention and**	See Dimension 2 above. In addition, the effectiveness review states that, 'It is important to note that the pathway from improved knowledge and information to changes in attitudes towards vaccination and, finally, to improved uptake of vaccination is not necessarily linear or simple. Increased	*Unclear or unable to assess*	Insufficient information to make an assessment. However, it is likely that the pathways are variable and long.

**the outcome it is intended to effect**	knowledge may, for example, result in more informed decision-making among caregivers, but not in increased childhood vaccination uptake.’ [1] Neither of the reviews attempted to construct a causal pathway or logic model based on the review findings.		

References

1. Saeterdal I, Lewin S, Austvoll-Dahlgren A, Glenton C, Munabi-Babigumira S: **Interventions aimed at communities to inform and/or educate about early childhood vaccination**. *The Cochrane database of systematic reviews* 2014(11):CD010232.
2. Ames HMR, Glenton C, Lewin S: **Parents' and informal caregivers' views and experiences of communication about routine childhood vaccination: a synthesis of qualitative evidence**. *Cochrane Database of Systematic Reviews* 2017(2):CD011787.
3. Andersson N, Cockcroft A, Ansari NM, Omer K, Baloch M, Ho Foster A, Shea B, Wells GA, Soberanis JL: **Evidence-based discussion increases childhood vaccination uptake: a randomised cluster controlled trial of knowledge translation in Pakistan**. *BMC Int Health Hum Rights* 2009, 9 Suppl 1:S8.
